# Effect of Physico-Chemical Properties of Nanoparticles on Their Intracellular Uptake

**DOI:** 10.3390/ijms21218019

**Published:** 2020-10-28

**Authors:** Parinaz Sabourian, Ghazaleh Yazdani, Seyed Sajad Ashraf, Masoud Frounchi, Shohreh Mashayekhan, Sahar Kiani, Ashok Kakkar

**Affiliations:** 1 Department of Chemical and Petroleum Engineering, Sharif University of Technology, Azadi Ave., Tehran 11155-9465, Iran; parinazsabourian@gmail.com (P.S.); gh.yazdani97@yahoo.com (G.Y.); sajadashraf00@gmail.com (S.S.A.); mashayekhan@sharif.edu (S.M.); 2 Department of Chemistry, McGill University, 801 Sherbrooke St. West, Montréal, QC H3A 0B8, Canada; 3 Department of Brain and Cognitive Sciences, Cell Science Research Center, ROYAN Institute for Stem Cell Biology and Technology, ACECR, Tehran 16635-148, Iran; sahar_kiani@royaninstitute.org

**Keywords:** intracellular delivery, nanoparticles, physico-chemical properties, cellular uptake, active/passive transport

## Abstract

Cellular internalization of inorganic, lipidic and polymeric nanoparticles is of great significance in the quest to develop effective formulations for the treatment of high morbidity rate diseases. Understanding nanoparticle–cell interactions plays a key role in therapeutic interventions, and it continues to be a topic of great interest to both chemists and biologists. The mechanistic evaluation of cellular uptake is quite complex and is continuously being aided by the design of nanocarriers with desired physico-chemical properties. The progress in biomedicine, including enhancing the rate of uptake by the cells, is being made through the development of structure–property relationships in nanoparticles. We summarize here investigations related to transport pathways through active and passive mechanisms, and the role played by physico-chemical properties of nanoparticles, including size, geometry or shape, core-corona structure, surface chemistry, ligand binding and mechanical effects, in influencing intracellular delivery. It is becoming clear that designing nanoparticles with specific surface composition, and engineered physical and mechanical characteristics, can facilitate their internalization more efficiently into the targeted cells, as well as enhance the rate of cellular uptake.

## 1. Introduction

The past two decades have witnessed the development of state-of-the-art nanotechnology, with the emergence of its demonstrated potential in biosciences [1]. Nanoparticles (NPs) that have at least one dimension ranging from 1 to 1000 nm, and can carry biologically active molecules, have provided an advantageous platform in biomedical applications, including imaging and intracellular delivery of bioactive agents [2,3]. This is generally related to the unique NP characteristics such as size, high surface ratio, stability in physiological conditions, controlled drug delivery, and low probability of invoking immune response [4]. In order to achieve a safe and efficient delivery of drug-loaded NPs into the cells, detailed knowledge of their interactions with cell surfaces is essential. NP–cell interactions influence diverse pathways in cellular uptake. Electroporation and microinjection can forcefully drive NPs into the cells. However, use of these methods can lead to cell membrane deformation and destruction, and is thus limited [5]. Cell membrane pores induced by electroporation, cause permanent permeability that leads to cell death [6], and microinjection results in physical damage to cell membrane [7].

**Scheme 1:**
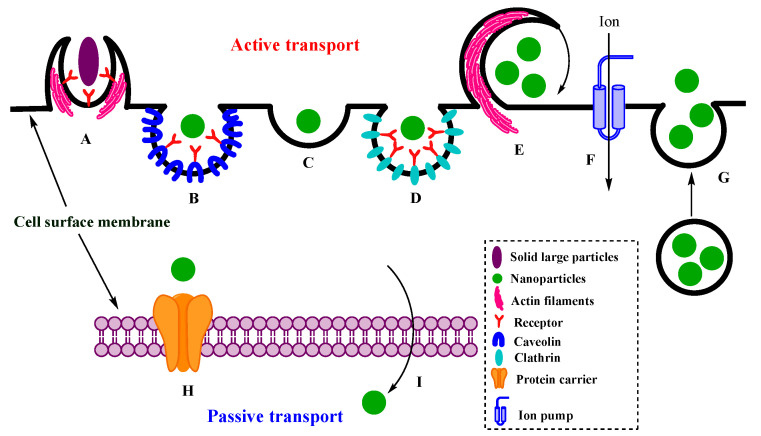
Active and passive cell uptake of particles: (**A**) phagocytosis, (**B**) caveolin-mediated endocytosis, (**C**) clathrin–caveolin-independent endocytosis, (**D**) clathrin-mediated endocytosis, (**E**) macro-pinocytosis, (**F**) ion pumps, (**G**) exocytosis, (**H**) facilitated diffusion, and (**I**) simple diffusion.

Before NPs enter into the diseased/targeted cells, they must pass through primary barriers such as epithelia of the skin, gastrointestinal tract (GI) or blood circulatory system. For specific cells that require excess protection, NPs must pass through secondary barriers including the blood brain barrier (BBB), blood spinal cord barrier (BSCB), blood testis barrier (BTB) and placenta [8,9]. There are several pathways of cellular uptake of NPs, molecules, and ions. For example, small non-polar molecules diffuse freely through cell membranes in the direction of concentration gradient (passive transport) [10], and can also go through facilitated diffusion and osmosis. Amino acids and ions go through the plasma membrane by integral membrane proteins or ion channels (active transport) [11]. Active transport pathways include endocytosis, exocytosis and ion pumps, and channels [12]. Endocytosis is the main method of bio-macromolecules internalization into the cells. Since NPs are similar in size to those of bio-macromolecules, they often internalize into the cells by endocytosis pathway [13,14]. Scheme 1 shows different pathways of cellular uptake including passive and active transport.

We will first summarize various pathways of cellular uptake of different NPs including polymeric (soft), metal and silica-based, by emphasizing their active bulk transport into the cells via different endocytosis methods. Material composition of each NP can be representative of its mechanical properties, which affect intracellular uptake. The cellular uptake of soft, metallic and silica NPs, which have varied stiffness, is evaluated collectively here to determine the effect of their mechanical properties. Soft NPs, including lipidic and polymeric, can be deformed much easier than metal and silica NPs, and should be able to overcome a higher energy barrier, and thus a lower cell uptake. Subsequently, several parameters that influence intracellular delivery, including size and shape of NPs, lipid/protein corona, and surface modifications, including surface electrical charge, hydrophobicity and conjugated ligands, are presented. The optimized physical, chemical and mechanical properties of NPs can facilitate both cell uptake and cargo delivery into the cells. It is worth mentioning that all these parameters influence cell–NP interactions, and NP transport into the cells. Several studies have shown that the physiological characteristics of each cell type and the surface chemistry of NPs play a critical role in cell–NP interaction [15]. For example, to enhance the efficiency of targeted cell uptake and drug delivery, it is desired to design NPs with a negative surface charge, to prevent the accelerated blood clearance (ABC). The latter isa phenomenon in which molecules and particles upon administration can be rapidly cleared from the blood, by the kidney and liver [16,17]. In contrast, a positive surface charge of NPs increases cell affinity and uptake in diseased tissues, by the enhanced permeability and retention (EPR) effect. To increase intracellular drug release, stimuli-responsive nanocarriers are widely used to efficiently deliver therapeutics in response to the biological stimuli, such as reactive oxygen species (ROS), pH, temperature, etc. Stimuli-responsive NPs can be designed by ligand binding or modifying surface chemistry of NPs [18]. In this review, we take a convergent approach to obtain a detailed understanding of a key tenet of effective therapeutic interventions, in a single venue. We begin with a brief synopsis of the intracellular transport pathways, and subsequently bring into focus the role played by the physicochemical properties, including size, shape, surface chemistry, ligand binding, and mechanical attributes of a variety of nanoparticles, on their intracellular fate.

## 2. Intracellular Transport of NPs

### 2.1. Active Transport

#### 2.1.1. Endocytosis

Endocytosis is an energy-dependent pathway in which cell membrane deformation and its wrapping around the introduced particle takes place (Scheme 1). The chemical composition of NPs as well as the functionalities on the cell surface both play crucial roles in cellular uptake of NPs. Specifically, size, shape, surface electrical charge and hydrophobicity of the NPs and cell are key factors in NP–cell interactions and further endocytosis of nanocarriers [19].

Size of the NPs is the parameter that classifies endocytosis pathways into two subgroups: micro- and nanoscale. Microscale endocytosis includes phagocytosis and macro-pinocytosis, which are responsible for engulfing NPs larger than 500 nm. Nanoscale endocytic pathways, such as clathrin-mediated, caveolin-dependent and clathrin/caveolin-independent endocytosis, are desired for smaller particles [20].

**Scheme 2:**
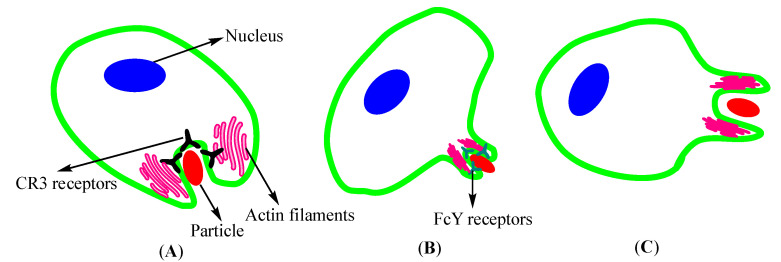
Different mechanisms of nanoparticle (NP) phagocytosis: (**A**) CR3-mediated phagocytosis occurs by sinking NPs through CR3-receptors; (**B**) zipper mode phagocytosis via Fcγ-receptors including cell progression; (**C**) trigger mode phagocytosis with no receptors takes place through stimulating ruffles around particles.

**Phagocytosis.** Phagocytosis occurs by professional and non-professional phagocytes, which are distinguished from each other by the lack or presence of efficient receptors [21]. Generally, this process happens by deformation of cell membrane and engulfing the particle, leading to phagosome formation. Scheme 2 presents different mechanisms of NP phagocytosis. In mammals, it is primarily carried out by professional cells such as macrophages, monocytes, and neutrophils and through receptors including immunoglobulin (fragment-crystallizable region receptor, Fcγ), and complement C3bi (CR3) receptors (Scheme 2A, integrin αMβ2, Mac1) [22].

In Fcγ-mediated phagocytosis, also known as “*zipper-mode*”, particles bound to immunoglobulin G (IgG) attach to cell membrane by Fcγ (Scheme 2B). This process stimulates actin polymerization and membrane extension over the introduced particle, leading to more ligand–receptor interactions and further engulfment of particles. Actin polymerization continues until complete zipping of the particle by membrane, and phagosomes formation [23]. Phagocytosis mediated by CR3, consisting of α and β-integrin chains, differs from zipper-mode. In CR3-mediated phagocytosis, phagosome walls are less adherent to the particle than in zipper mode, therefore, particles sink into the cell. There is another type of phagocytosis called “*trigger mode*” (Scheme 2C) that occurs through stimulating ruffles, similar to macro-pinocytosis [23,24].

**Macro-Pinocytosis.** The fluid phase internalization, which is called macro-pinocytosis and occurs on membrane ruffles, is one of the actin- and Ras-related C3 botulinum toxin substrate (Rac)1-dependent endocytosis [25]. In most cells, membrane ruffling is induced when the ruffles are folded back onto themselves, maintaining the particles trapped between their sites, and then large vesicles called macro-pinosomes are formed of sizes up to 5 µm [26] (Scheme 1E). Despite the other pinocytosis pathways, membranes of macro-pinosomes lack specific proteins such as caveolin and clathrin [27].

**Clathrin-Mediated Endocytosis.** Most pinocytic pathways, including clathrin-mediated endocytosis, proceed via ligan–receptor interactions. The ligand–receptor complex gets entrapped inside the vesicles coated by cytosolic proteins (mainly clathrin). Clathrin is made up of three heavy chains bound tightly to the backbone. Under certain circumstances and with assistance from the assembly proteins (APs), the clathrins form a cage around the vesicles. Dynamin formation around vesicle neck helps them pinch off from the membrane [28]. At first, clathrin-mediated endocytosis (Scheme 1D) was recognized by its important role in internalization of some essential nutrients, including cholesterol, carried by lipoproteins, and iron by transferrin [29]. For some NPs, clathrin-mediated endocytosis is the most prevalent type of cell uptake. However, internalization pathways depend on cell type and NP modifications. For example, polyethylene glycol (PEG)-poly lactide (PLA), chitosan, silica-based and poly(lactic-co-glycolic acid) (PLGA) NPs undergo clathrin-mediated endocytosis as their prominent pathway [30].

**Caveolin-Mediated Endocytosis.** Caveolae are typically 50 to 80 nm invaginations on cell membrane, rich in glyco-sphingolipids (GSLs), cholesterol, and lipid-anchored membrane proteins, mainly caveolin-1 subgroup. Caveolins, a subgroup of integral membrane proteins bind to cholesterol, and lead to caveolae formation [31,32]. However, the exact pathway is still not known, and investigations on polyomavirus simian virus (SV) 40 internalization show that after cell adhesion, which triggers membrane-fission reactions, caveolae pinches off from the membrane, and is released into cytoplasm [33]. One of the proteins participating in caveolin-mediated endocytosis, cavin, co-assembles with caveolin. It is responsible for membrane curvature, and then dynamin helps the vesicle to pinch off from the membrane [34]. One of the advantages of caveolin-mediated endocytosis is its ability to escape from converting to lysosomes and prevent the lysosomal degradation. When caveolae engulfs SV40, it ends up in caveosomes, but it does not fuse with lysosomes, and thus escapes from lysosomal degradation. However, in some cases ligands engulfed by caveolae can also be targeted to the late endosomes/lysosomes [35].

**Clathrin–Caveolin-Independent Endocytosis.** Clathrin–caveolin independent pathways can be divided into two subclasses, dynamin-dependent and dynamin-independent pathways. Dynamin-dependent pathway requires small guanosine triphosphatase Ras homolog A (GTPase RhoA) to uptake the molecules and NPs. It is responsible for internalization of β-chain of the interleukin-2 (IL-2R-β), Ɣc-cytokine and IgE receptors. This pathway could be prevented by RhoA inhibitors, due to the remarkable role of RhoA in regulating actin cytoskeleton dynamics, and recruiting actins to trigger the endocytosis [36]. It appears that dynamin-independent pathways are influenced by small GTPase, either by the cell division control protein (CDC) 40 or ADP rebosylation factor (ARF) 6. While vesicles of clathrin-mediated endocytosis are small and spherical, vesicles of CDC42 endocytosis are long, wide and responsible for the fluid-phase internalization in various cell types. The direct role of ARF6 in endocytosis pathway is unknown. The mechanisms of other types of dynamin-dependent and dynamin-independent pathways are not still investigated [37].

On the other hand, there are endocytic pathways other than clathrin and caveolae-dependent, which occur through cholesterol and sphingolipids rich membrane domains of cells. It has been shown that both caveolae and glycolipid rafts are dynamin-dependent and sensitive to cholesterol depletion. Glycolipid rafts can internalize ligands without caveolin-1, which is a key factor in the flasked-shape morphology of caveolae on membrane surface [38].

#### 2.1.2. Exocytosis

Exocytosis occurs in all types of cells either to export substances from cell cytosol to extracellular space, or to add new components to the cell membrane (Scheme 1G). “Constitutive” and “Regulated” exocytosis are two main pathways for exocytosis and, in both, molecular motors carry the vesicles to export them to the cell membrane. Constitutive exocytosis occurs when the vesicles reach the cell membrane and the fusion is not controlled by a known mechanism. On the other hand, in regulated exocytosis, vesicles accumulate near the membrane until a signal reaching the cell membrane causes the cytosolic messenger substance to appear and start the conformation in the cell membrane for the exocytosis [39]. After internalization, NPs can be trapped in lysosomes and later go through exocytosis. There are some factors that influence NP exocytosis including cell type, NP physico-chemical properties, NP concentration and cellular incubation time, distribution of NPs into organelles, accelerators and inhibitors of exocytosis process [40].

#### 2.1.3. Ion Pumps

The continuous flow of ions into and out of cells is necessary for cell survival and function, and ion pumps maintain the gradients across the cell (Scheme 1F). Selective transportation of ions such as sodium, potassium, calcium, hydrogen and a few others can occur through ion pumps [12]. Pumps use adenosine triphosphate (ATP) or light to drive the ions against their electrochemical gradient. P-type ATPases and the ATP-binding cassette pumps, two types of ATP dependent pumps, bind to ATP and transport the bound ion across the membrane [41].

The most investigated are sodium–potassium (Na^+^–K^+^) pumps, which drive the Na^+^ ions outside and K^+^ ions into the cell and are responsible for keeping the concentration gradient of ions and negative potential of cells interior constant. The concentration of calcium ions in most cells is very low compared to extracellular fluid and calcium pumps hold this concentration gradient constant. One of the two types of calcium pumps is presented on the cell membrane and pumps calcium ions outside the cell. The second type pumps the calcium ion inside the cells or more vesicular organelles (such as mitochondria and muscle sarcoplasmic reticulum) [42].

### 2.2. Passive Transport of NPs

Passive transport or diffusion is an uncompetitive movement of molecules and NPs, either directly through membrane phospholipids or in combination with membrane proteins. Since diffusion is down the concentration gradient, it needs no energy. Passive uptake is divided into simple and facilitated diffusion (Scheme 1). In simple diffusion, the molecules or NPs enter the cell by passing through intramolecular spaces in membrane or protein channels. Lipophilic agents directly pass through lipid bilayer, but hydrophilic ones internalize by protein channels. In both pathways, the internalization rate is proportional to the concentration of diffusing substance [43]. The second passive pathway, facilitated diffusion, requires certain protein carriers. In the facilitated pathway, the rate of internalization is at maximum when the concentration of diffusing substance increases [42].

## 3. Role of Physico-Chemical Properties of NPs in Cell Uptake

There are several nanoparticle parameters that influence their uptake into the cells [44,45,46,47,48,49,50,51], and some recent studies exemplifying these are shown in Table 1. A brief overview of NP physicochemical properties and their impact on internalization is described below.

### 3.1. Size

The primary factor in designing NPs passing through the cell membrane is their size (Table 1), which can range up to hundreds of nanometers [5]. NPs enter the cell via passive or active pathways, but their size can influence the internalization mechanisms, cargo delivery and immune response [44,45]. For example, in all receptor-mediated endocytosis pathways, the key factor for initiation of membrane wrapping is activation of receptors on the targeted cells by specific ligands on the surface of the NPs. Increasing ligand density on the nanoparticle surfaces requires a larger size and higher aspect ratio, which is the ratio of characteristic length to diameter of NPs. If the size of spherical NPs is smaller than 30 nm, they are not able to drive the engulfment process. On the other hand, those above 60 nm can cause steric hindrance and receptor saturation. Most in vitro studies have indicated that the size range from 10 to 60 nm, regardless of the NP composition and surface charge, is the most optimum diameter for their cellular uptake [52].

As an example of the effect of size, cellular uptake of latex particles in the range of 50 to 500 nm was investigated in non-phagocytic cells. Fluorescently labelled microspheres were incubated with mouse melanoma B16 cells. In the processes that inhibited clathrin-mediated endocytosis, either by inducing potassium deficiency in cells or pre-treating with chlorpromazine, it was noted that the NPs smaller than 200 nm entered cells preferentially by clathrin-mediated endocytosis, while the larger ones by the caveolae-mediated pathway [53]. Similarly, uptake of spherical polyethylene glycol-modified TiO_2_ (TiO_2_-PEG) NPs into NCI-H292 cells was also found to be size dependent. After incubating cells in hyperosmotic sucrose to prevent clathrin-mediated endocytosis, it was noted that the uptake of 100 and 200 nm (in contrast to 300 nm) NPs decreased [54].

**Figure 1:**
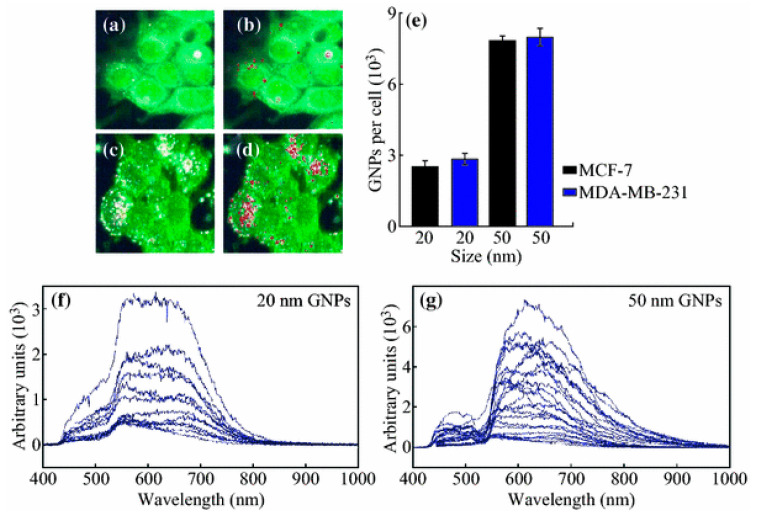
Uptake of gold nanoparticles (GNPs). Hyperspectral image of cell uptake of (**a**) 20-nm- and (**c**) 50-nm-sized GNPs; (**b**,**d**) GNPs clusters mapped using reflectance spectra of GNPs; (**e**) GNP internalization per cell for 20- and 50-nm-sized GNPs in two different cell lines. (**f**,**g**) The reflectance spectra of the 20- and 50-nm-sized GNPs in the monolayers (**a**,**c**). Reprinted with permission from [55]. Copyright 2016 Springer Nature.

Due to the growing interest in gold nanoparticles (GNPs) for biomedical applications including intracellular imaging, photo-thermal therapy and drug delivery, their functionalization and interactions with cells have been widely investigated. As shown in Figure 1, the cellular uptake of GNPs was found to be size dependent, as the 20-nm-sized GNPs had lower cellular uptake, compared to 50 nm, in both MCF-7 and MDA-MB-231 tumor cell lines. This was due to the fact that smaller GNPs have less surface area to interact with the cell membrane receptors, and, as a result, cells provide lower energy for engulfing NPs [55]. Several studies have now reported that 50 nm is the optimum size for cell internalization [56].

The intracellular uptake of 14, 50 and 74-nm GNPs by HeLa cells also showed maximum cellular uptake of 50-nm-sized NPs. The pathway used for internationalization is not fully understood, but it was suggested that it could possibly be endocytosis [57]. In another report, it was noted that the cell uptake of single GNPs in the size range of 4 to 17 nm increased due to their higher passive transport [58]. It has been suggested that the cellular uptake of functionalized GNPs changes inversely with the particle size, and smaller GNPs show higher levels of internalization than the larger ones. The disparity in results of these studies might be due to the variation in ligands and preparation methods for the functionalized GNPs [56,59,60].

Since red blood cells (RBCs) lack endocytic system, these are ideal candidates to study passive internalization of NPs [61,62]. To illustrate the size effects on passive transport, an analysis of RBCs was performed, using polystyrene (PS) NPs containing different surface ligands and charges. It was found that only their size influenced the efficiency of uptake by RBC. However, the overall cell uptake of NPs was too low (around one particle per cell) [63].

It should also be noted that the size of NPs changes as they are passing through the biological environment. Therefore, to study the nanoparticle size effect on cellular uptake, the sizes of both the original and altered sizes after NPs exposure to the biological medium should be considered [64]. For instance, protein corona, which will be discussed later in this review, is one of the key factors that changes the NP size after exposure [65].

### 3.2. Shape

Nanoparticles come in various shapes/geometries including spherical, rod-like, discoid, clubbed and nano-needle, and it is becoming clear that the NP morphology has a crucial role in their circulation, bio-distribution, targeted delivery and cellular uptake [46,66,67]. NPs with higher aspect ratio show greater affinity to enter the cells by either active or passive transports. Carbon nanotubes (CNTs) with cylindrical geometry and high aspect ratio could easily penetrate through the cell membrane [5], and it has been shown that the rod-shaped NPs have more accessible binding sites [68]. It suggests that nanoparticle–cell interactions in these NPs occur more efficiently and bring extensive uptake compared to spherical NPs with the similar size [69].

**Figure 2:**
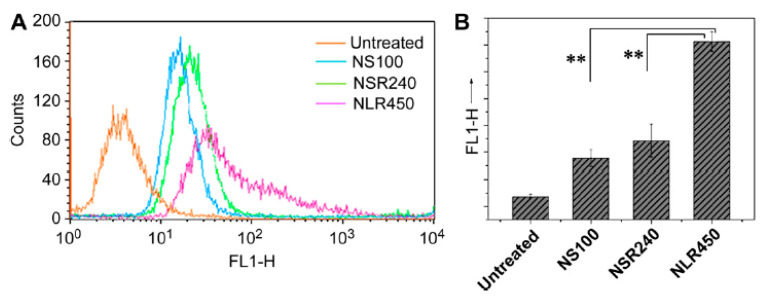
Quantification of the internalization of mesoporous silica NPs: fluorescence intensity (**A**), particle numbers (**B**), using fluorescent-activated cell sorting (FACS). ** Indicates statistical significance, *p* < 0.01). Reprinted with permission from [70]. Copyright 2010 Elsevier.

The effect of the shape of mesoporous silica nanoparticles on cell uptake by human melanoma cells (A375) was studied by Huang et al. As shown in Figure 2A,B, spherical NPs with the mean size of 100 nm (NS100) showed less fluorescent intensity, compared to short and long rod-shaped NPs with the size of 240 (NSR240) and 450 (NLR450) nm, respectively. In addition, the number of 450-nm-sized NPs internalized into the A375 cells was much higher than those of 100 and 240 nm. Rod-shaped NPs internalized more efficiently into the cells, due to their higher aspect ratios and contact surfaces to adhere to the cell membranes [70]. However, the time needed for wrapping is also a factor that influences the rate of internalization. Since the wrapping time for elongated NPs is longer than for spherical ones, their uptake rate can be less than nanospheres [71]. It has been shown that discoid NPs have higher internalization into Hela cells than rod-shaped NPs with the similar aspect ratio [69]. In another study, the comparison between the cell uptake of spherical and cylindrical NPs, prepared by self-assembly of polyacrylic acid (PAA) and PS diblock copolymer, had a lower internalization rate of spherical NPs [72,73].

### 3.3. Corona

**Figure 3:**
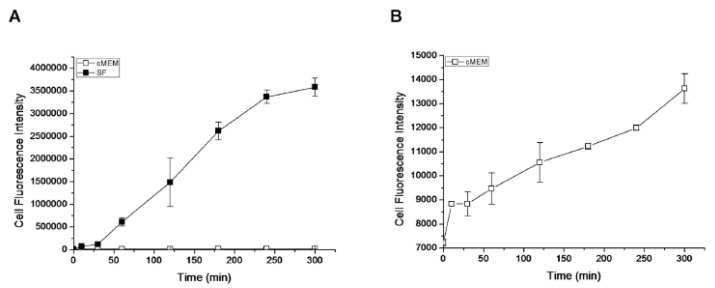
(**A**) Kinetics of cell uptake of fluorescently labeled silica NPs (25 μg/mL) in the complete (cMEM) and serum-free (SF) media, measured by flowcytometry; (**B**) curve in cMEM from A alone. Reprinted with permission from [77]. Copyright 2012 American Chemical Society.

When a nanoparticle comes in contact with the biological fluid, it experiences changes in its nature, surface structure and interactions with the cells. Biomolecules in biological fluids can be adsorbed on the surface of NPs and form a layer (corona) around them [74]. The cellular uptake of NPs, which are in contact with particular cells, can be different in the presence and lack of corona of a specific composition [75,76]. It has been demonstrated that the internalization rate of silica NPs in the serum-free biological conditions is higher than the rate of their uptake in the presence of serum (Figure 3). The proteins adsorbed on the surface of NPs after incubation with cells were mainly cytoskeleton and membrane proteins. This is indicative of the tendency of the nanoparticle to reduce its surface energy by binding to the proteins, and developing strong cell–NP interaction [77].

**Scheme 3:**
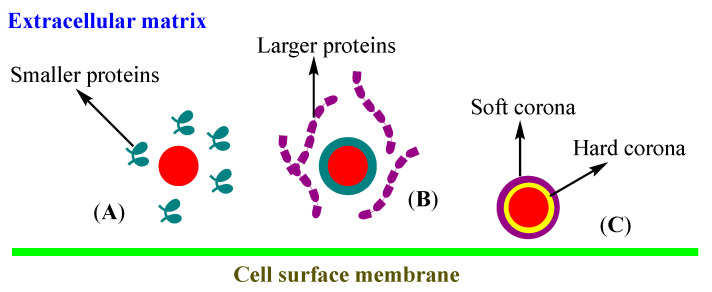
Protein corona formation on the surface of NPs: (**A**) adsorption of smaller proteins to NP surface by rapid diffusion, (**B**) replacement of small proteins by larger ones, reconfiguration of proteins and final hard and soft corona formation (**C**).

As shown in Scheme 3, two types of corona have been defined based on their structure: “hard” which can be formed when the biomolecules are irreversibly and directly bound to the NP surface, whereas “soft corona” consists of biomolecules that are reversibly bound to the hard corona or NP surface [47,78,79]. The lifetime of the hard corona has been shown to be several hours and it can define the NP surface properties. Since the biomolecules compete for the limited surface on NPs, at first NPs will be covered by more abundant small molecules, and subsequently replaced by the larger and high affinity molecules over time, to form the hard corona [80,81].

Lipoproteins are formed when lipids bind to the apo-lipoproteins to facilitate their transportation. NPs can bind to the lipoproteins as a corona structure [82]. Recent investigations have shown that the NPs not only bind to the apo-lipoproteins but also interact with the lipoprotein [83].

One of the major challenges for targeted delivery of NPs is their cellular uptake by phagocytes [84]. In a biological environment, NPs can be surrounded by various types of biomolecules including proteins of the extracellular matrix, serum albumin, apo-lipoproteins, complement components and immunoglobulins which will form the corona. The receptors expressed on the phagocytic cell membranes attach to the proteins on the surface of NPs and internalize them [84,85]. Even if the phagocytes do not uptake NPs, the corona layer covers the ligands, and the NPs are not recognized by the targeted cells, and lose their specificity [86]. One of the most common and efficient solutions for this problem is coating NPs with a barrier-like layer composed of polyethylene glycol (PEG), called PEGylation. PEG chains on the surface of NPs create steric hindrance, which inhibits the protein adsorption and decreases recognition by macrophages [87].

One of main goals in targeted delivery is to internalize NPs into specific cells by an endocytosis pathway such as clathrin, caveolin, etc. Studies on targeted drug delivery have shown that NPs lose their specificity when coated with corona. Corona not only changes the surface composition and structure of NPs, which directly influences the cell–NP interactions, but also affects the geometry and size of NPs, which play a crucial role in cellular uptake [88].

### 3.4. Surface Chemistry

#### 3.4.1. Surface Electrical Charge

Surface charge of the NPs can be tuned by conjugating functional groups [48]. Since the electrical charge of the cell membrane is negative, the positively charged NPs are easily attracted to the cellular membrane, and internalized mostly by endocytosis pathways [89]. Negatively charged NPs are expected to be uptaken much less than neutral ones, but several reports have shown the inverse results [13]. For example, a study of carboxy methyl substituted dextran-coated NPs with a surface charge between −50 and +5 mV and incubated with Caco-2 human colon cancer cells, suggesting internalization, despite higher negative charge. Using inhibitor assays, non-specific internalization pathways for most negatively charged NPs were observed [90].

In summary, NPs adsorb proteins on their surfaces in biological medium, which later determine their fate in the cellular microenvironment [91]. Density and type of adsorbed proteins are influenced by the surface charge and the type of functional groups on NPs. Using PS NPs modified with either basic or acidic functional groups, it has been shown that cationic NPs bind to the plasma proteins, which have isoelectric points (pI) < 5; whereas anionic NPs bind to the proteins with pI > 5. It was also suggested that the adsorption of the protein species on the surface of NPs depended on the surface charge density. For instance, both IgG and albumin prefer binding to the NPs with strongly basic (NH_2_) or weakly acidic (COOH) groups [92]. Due to the effect of IgG among other proteins on particle removal from the blood, its adhesion to the surface of NPs influences the in vivo cellular fate of NPs [93].

#### 3.4.2. Hydrophobicity and Hydrophilicity

Hydrophobicity of NPs has a remarkable effect on NP–cell interactions. Several studies have suggested that surface hydrophobicity of NPs affects not only their cell uptake, but also their opsonization and bio-distribution [49,65,94]. Core-shell NPs with hydrophobic PLGA surface had enhanced cell uptake, and this composition also supported bio-distribution of NPs in the eye tissues [95]. Silver (Ag) NPs functionalized with hydrophobic copolymers of di (ethylene glycol) methyl ether methacrylate and oligo (ethylene glycol) methyl ether methacrylate (OEGMA) have been prepared, and the NPs with various copolymer ratios showed different behavior related to surface hydrophobicity. In the presence of serum proteins, hydrophobicity of NPs increased and, subsequently, their cellular uptake and cytotoxicity increased. However, cellular internalization was less dependent on hydrophobicity in serum-free medium, suggesting the critical role of corona formation on the cell response [94].

Corona formation plays a crucial role in NP–cell interactions and cell uptake, since it changes the nature and chemistry of NPs surface. For instance, methoxy-PEG modified with a thiol group (mPEG-SH) is known to be a very beneficial surface modification for NPs due to its resistance to protein adsorption and low toxicity. However, it has been shown that mPEG-SH layer on the surface of NPs can be substituted with cysteine molecules of proteins, and then NPs removed by macrophages. This problem can be resolved by adding a hydrophobic shield between the NP surface and PEG layer [96]. The synergic effects of NP hydrophobicity and corona formation on cell uptake have been demonstrated by designing zwitterionic NPs with tunable hydrophobicity. Cellular uptake of these NPs improved by increasing their surface hydrophobicity in serum-containing and serum-free media. This suggests that the NP hydrophobicity influenced cell uptake indirectly by affecting the corona formation process [97].

Hydrophobic and hydrophilic NPs interact with cells through different mechanisms. Molecular dynamic (MD) simulations have suggested that hydrophobic NPs enter the lipophilic core of cell membranes, while semi-hydrophilic NPs can be adsorbed on the surface of the membrane bilayer. These results indirectly demonstrate that endocytosis is the main mechanism for cellular uptake of semi-hydrophilic NPs [98]. In another study of similar nature, hydrophobic NPs were shown to enter cells by directly penetrating into the cell membrane. However, hydrophilic NPs were adsorbed on the bilayer surface, and passed through the cell via membrane wrapping [99].

#### 3.4.3. Ligand Binding

Recently, a study compared the cellular internalization of NPs with different core materials (Pt, Pd and Au) and surface ligands, and it showed that both ligands and core materials affect the surface hydrophobicity of NPs and cell uptake. It was observed that Pd and Au NPs, which were more hydrophobic than Pt, displayed higher cell uptake. It was 10 to 15 times lower for the Pt NPs than Au and Pd NPs, but these still entered the cells in a very small amount via non-specific phagocytosis and pinocytosis [100]. Modifying NPs with ligands has been used widely for various goals, including increasing circulation time and cell uptake [65], target delivery and reducing cytotoxicity [50,101].

One of the obstacles for the NP to reach the targeted site is its clearance by immune system. Accelerated blood clearance (ABC) of NPs is a natural function of kidney, liver and immune system, and it removes the excess amounts of drugs and drug-loaded NPs from the blood. In order to increase the circulation time and therapeutic efficacy, NPs are functionalized with certain ligands or biological molecules. Ligands bonded chemically or physically to the surface of NPs inhibit biological proteins to adhere to their surfaces and prevent the immune system and liver from clearance and opsonization of NPs. This can be achieved by utilizing NPs containing specific peptides [102], membranes derived from blood cells [103], and also by the addition of serum proteins to drugs [104]. For instance, the membrane protein CD47 is one of the proteins that aids phagocytes to distinguish the “*self*” cells from foreign particles and cells, and it has been shown that NPs functionalized with these peptides show decreased macrophage-mediated clearance and enhanced drug delivery to tumors [102].

Coating NPs with membranes of specific cells aids them in avoiding clearance by immune system. Nanoporous silicon-based particles coated with membranes derived from leukocytes (white blood cells of the immune system) reduced and delayed NP uptake by mononuclear phagocyte system, by avoiding opsonization. Furthermore, non-coated NPs were bound to normal and inflamed endothelial cells in the same amounts, and coated NPs to inflamed endothelial cells two times more than healthy cells, which seems to be related to lymphocyte function-associated antigen 1 (LFA1) present on the leukocyte membranes [103]. Another method of NP modification for enhanced targeting and extended circulation time is the addition of serum proteins, such as albumin. Using albumin as a ligand for paclitaxel NPs, it has been shown to transport NPs across endothelial cells and results in accumulation of NPs in tumor sites. The albumin binds 60-kDa glycoprotein (gp60) receptor (albondin) on endothelial cells, which is followed by caveolin-1 recruitment, appearance of membrane invagination around paclitaxel NPs, and NP internalization. Evidence shows that tumor uptake of albumin is much higher than healthy tissue, and this might be due to the secretion of SPARC (secreted protein, acidic and rich in cysteine), an albumin-binding protein in tumors [104].

In a bid to examine the role of ligand mobility, cellular uptake of PLA-PEG nanoparticles bound to angiotensin-II was investigated using rat mesangial cells. It was found that a lower ligand density (20%) allowed more ligand mobility and enhanced binding to specific receptors, increasing it to 80%, and decreased cellular uptake, which may be associated with lower free movement of NPs [105].

While the main goal of NP surface modification with ligands is to enhance drug delivery efficacy, some reports have studied the effect of ligand distribution on cellular uptake. Using the statistical dynamics model of endocytosis, it has been shown that when the receptor density is sufficient, the uniform distribution of ligands on NP surface has the maximum cellular uptake. The uniform distribution of ligands has also been observed on viral capsule, but it has not yet been proven if this characteristic guarantees virus internalization into the host cell [106]. NPs also show higher uptake and membrane wrapping speed when they have homogeneous ligand distribution. This could be explained with the low activation energy needed to wrap the homogenous NPs. The ligand free patches slow down the uptake process, and if the patches are bigger than 20% of the surface, the cellular uptake will be inhibited [107].

### 3.5. Mechanical Properties

Quantifying the effect of NP elasticity on cellular uptake is challenging because of experimental difficulties in measuring the micro- and nano-scale mechanical properties of NPs [51,108]. One of the problems in the study of the elasticity effects on the NP endocytosis is the fact that many physiochemical properties of NPs are strongly related to each other. This issue makes it hard to synthesize NPs with an exclusive parameter [109]. The elasticity of NPs can affect their bio-distribution, targeted delivery and cellular uptake in a physiological environment. The theoretical models on cellular uptake of NPs have helped predict that their wrapping by plasma membrane is energetically less desired for “soft” NPs than “stiff” ones. These models demonstrate that stiff NPs have higher rates of cellular uptake because of their easier membrane penetration and larger surface area to interact with the cell plasma membrane. “Stiffness” can be measured by a physical parameter, “*Young’s modulus*”, which defines the correlation between tensile stress and strain for a given material [108,110,111].

To measure the stiffness and topology of NPs, atomic force microscopy (AFM) can be employed [112]. It enables one to determine the quantitative and qualitative analysis of different properties of NPs such as morphology, size, surface roughness and composition. It also provides details about different geometries of NPs, and their physical properties such as magnetic behavior. AFM has been shown to be efficient for the study of soft and hard materials, regardless of their conductivity and opaqueness. Topography of NP surfaces forms different peaks with various color gradients or grayscale. Therefore, a multicolor topology image of sample surface helps to identify and measure the mechanical parameters of NPs [113].

**Figure 4:**
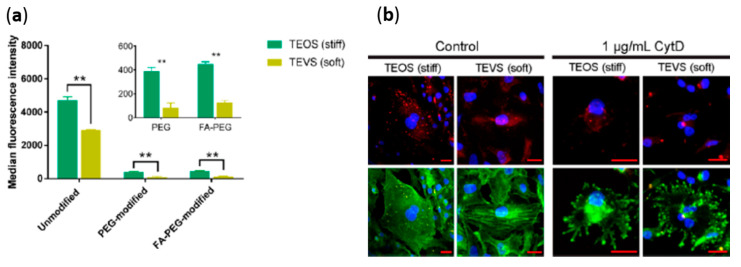
Nanocapsular stiffness effects: (**a**) cellular uptake by RAW264.7 murine macrophages of surface-modified NPs; (**b**) fluorescence micrographs showing the cellular uptake of FA-PEG-modified NPs by CytD-treated SKOV3 cells. (** Indicates statistical significance, *p* < 0.01). Reprinted with permission from [114]. Copyright 2018 American Chemical Society.

In a recent study, nano-liposomes (NLPs) containing alginate were prepared, in which concentrations of calcium alginate to control the cross-linking efficiency of NLPs were varied. Controlling the cross-linking efficiency allowed preparation of NLPs with different *Young’s moduli*. Alginate filled liposomes without crosslinking led to soft NLPs with *Young’s modulus* of ~1.6 MPa, whereas augmentation of cross-linking resulted in stiff NLPs with *Young’s moduli* up to 19 MPa. When these NLPs were exposed to the neoplastic and non-neoplastic cells (e.g., human mammary epithelial MCF10A), all cells engulfed the soft NLPs remarkably higher than the stiffer ones, due to the soft NPs being more fused into the cells along with their endocytosis internalization [112]. To measure the mechanical properties of polymeric nano-capsules using AFM, drug-filled silica nano-capsules were synthesized from two different silica precursors (tri-ethoxy vinyl silane (TEVS) and tetra-ethyl orthosilicate (TEOS)) (Figure 4). Based on AFM, TEVS nano-capsules exhibited lower stiffness than TEOS nano-capsules at different reaction times of 30, 40 and 50 h. TEVS nano-capsules prepared in a reaction time of 50 h displayed shell thickness of 24 nm and stiffness of 16.6 MPa. The stiffness of TEOS nano-capsules increased from 696 to 810.4 MPa as the shell thickness was raised from 12.5 to 24.8 nm, when the reaction time was prolonged from 30 to 50 h. Furthermore, by considering the nano-capsule as a uniform material, the *Young’s moduli* of the two structurally different nano-capsules were calculated to be 704 kPa and 9.7 GPa, using the “*Hertzian contact model”.* This study showed that cell uptake of stiff NPs (TEOS) was higher than soft NPs (TEVS) in both macrophages and tumor cells, due to the higher shape deformation of soft NPs [114].

In another investigation, AFM was used for measuring the *Young modulus* of NPs prepared by self-assembly of block copolymers, poly(1-*O*-methacryloyl-*β*-D-fructopyranose:1-*O*-MAFru) as the hydrophilic block, and polymethyl methacrylate (PMMA) with high glass transition temperature (T_g_), or poly (*n*-butyl acrylate: PBA), with a T_g_ below the room temperature, as the hydrophobic block. Calculation of the average modulus indicated that rod-like NPs prepared by poly(1-O-MAFru)-*b*-PMMA (*Young’s modulus* = 2500 MPa, ranging from 0.5 to 5 GPa) were stiffer than those prepared using poly(1-*O*-MAFru)-*b*-PBA (*Young’s modulus* = 350 MPa, ranging from 0.15 to 1.2 GPa). These values are comparable to the physical properties of the living nanoscopic organisms, namely viruses. Spherical viruses show *Young’s moduli* ranging from 100 to 2000 MPa based on their sizes. In addition, it was found that the stiffness of the virus varies with its maturation, and it influences its cellular internalization [115].

NP elasticity also affects their interactions with the immune cells. Polyethylene glycol diacrylate (PEGDA) NPs of the same size but different *elastic moduli*, around 10 kPa (soft NPs) and 3 MPa (stiff NPs), have been synthesized using a water-in-oil nano-emulsification method. In this study, the volume fraction of PEGDA was representative of NP elasticity. Differences between *elastic moduli* of soft and stiff NPs showed shorter blood circulation times for stiffer NPs due to their ABC performed by phagocytosis immune cells [116].

In addition to the above-mentioned methods, computational modelling has also been employed to simulate the penetration process of NPs with varied elasticity into the cells, using dissipative particle dynamics (DPD) technique. DPD is a mesoscopic simulation method that describes clusters of molecules moving together in a “*Lagrangian fashion*”, subject to soft quadratic programming. For instance, analysis by DPD simulation showed that the translocation abilities of hydrophilic NPs can be enhanced by increasing the NP stiffness, while the penetrability of hydrophobic NPs decreases in higher values of stiffness. It is due to the more efficient shape deformation of soft NPs than the rotation of stiff NPs during penetration across the cell membrane [117].

Through an analysis of some of the published reports on the effects of the mechanical properties of NPs on cellular uptake, it is noted that stiffer NPs internalize into living cells more efficiently than the softer ones. In contrast, there are several other studies that have reported exactly the opposite. As the nanomedicine continues to expand, more research dedicated to understanding the NP elasticity should be carried out, and hopefully these will improve our understanding of the NP–cell interactions.

## 4. Conclusions and Future Outlook

Physico-chemical properties of soft and metal nanoparticles play a vital role in disease diagnosis, treatment, and management. Considering there are numerous pathways for NP internalization into living cells, including active and passive transports, the influence of the changes in the properties of NPs, can help us understand cell–NP interactions, as well as the mechanism of their cellular uptake. Systematic and in-detail studies of these complex interactions are challenging, due to instability of NPs in biological environments. NPs are internalized into cells through both active and passive pathways, and covalent binding of ligands to modify particle surfaces has been shown to facilitate their active transport. Hydrophobic NPs go through the cells via simple diffusion. Positively charged NPs have more affinity to the cell surface, however, they undergo higher accelerated blood clearance than negatively charged NPs, which may be related to corona formation by oppositely charged serum proteins and lipids. Rod-like nanocarriers penetrate into the cells much easier than spherical NPs, due to their higher aspect ratio and surface area to bind to the proteins of cell membrane. Since F-actin is a structural protein of cells and senses the mechanical effects of the cell surface, stiffer NPs are internalized at a higher rate into the cell by F-actin performance, which poses a lower energy barrier. There are numerous factors such as NP aggregation/agglomeration, concentrations, cell type, pH, temperature, presence of ROS and varied mechanisms of cellular uptake. Through investigations of these, we are continuing to learn the important steps in delivering the therapeutic cargo. Much more still needs to be done to gain a detailed understanding of these important parameters. The exact mechanism of intracellular delivery of specific NPs with pre-determined physico-chemical properties is not fully understood. However, with the progress that has been made in the field of cellular internalization of NPs, we believe that a more detailed understanding of cell–NP interactions will be forthcoming in the near future. The latter will be crucial in recognizing structure–property relationships for the internalization pathways of inorganic, lipidic and polymeric NPs, into the living cells. In a similar vein, many factors such as cell type, NP administration methods, presence of physiological stimulus, including tissue acidity, temperature, free radicals or oxidants, enzymes, can significantly influence intracellular delivery of NPs. These factors need to be investigated to increase the cellular uptake rates of NPs for enhancing drug delivery.

## Figures and Tables

**Table 1:** Recent studies highlighting the influence of NP physicochemical properties on cellular uptake.

Physicochemical Property	Parameter	Nanoparticles	Cell Type	Uptake Mechanism	Important Features	Reference
Size	5–100 nm	Ag	B16	Clathrin-mediated endocytosis	Higher uptake of larger NPs; smaller cross plasma membrane faster	[45]
Shape	Nanospheres and nanostars	siRNA-conjugated-Au	U87 Glioblastoma	Endocytosis	Larger spheres (50 nm) and stars (40 nm) show higher uptake	[46]
Corona	Hard (HC) and soft (SC) corona proteins	Hard and soft corona modified silica and polystyrene	THP-1 and human brain endothelial	Endocytosis	Interactions of proteins on NP surface influence cell association	[47]
Surface charge	Positive, negative and neutral	PEG-*b*-PLA	Caco-2 and small intestinal epithelial	Clathrin and caveolin-mediated endocytosis	Positive charge improves uptake, transport, and distribution	[48]
Hydrophobicity	Nanogels	Amphiphilic polymeric system with varied hydrophibicity	Monocytic-like THP cells	Passive transport	Polymeric network hydrophobicity impacts protein binding and uptake	[49]
Ligand binding	Multivalent quantum dots 15–20 nm	Galactose-functionalized quantum dots	HepG2	Caveolae- and clathrin-mediated endocytosis	Galactose multivalency, a key factor in uptake mechanism	[50]
Mechanical properties	Polymer stiffness	Ganglioside (GM3)-functionalized lipid-wrapped PLGA-PLA	CD169- expressing macrophages	Actin-dependent phagocytosis	Core stiffness influences NP uptake and localization	[51]

## References

[1] Jahromi L.P., Panah F.M., Azadi A., Ashrafi H. (2019). A mechanistic investigation on methotrexate-loaded chitosan-based hydrogel nanoparticles intended for CNS drug delivery: Trojan horse effect or not?. Int. J. Biol. Macromol..

[2] Kumari N., Mathe V., Dongre P. (2019). Albumin nanoparticles conjugates binding with glycan-A strategic approach for targeted drug delivery. Int. J. Biol. Macromol..

[3] Soleimani M., Al Zaabi A.M., Merheb M., Matar R. (2016). Nanoparticles in Gene Therapy. Int. J. Integr. Biol..

[4] Kulkarni S.A., Feng S.-S. (2013). Effects of particle size and surface modification on cellular uptake and biodistribution of polymeric nanoparticles for drug delivery. Pharm. Res..

[5] Ma N., Ma C., Li C., Wang T., Tang Y., Wang H., Mou X., Chen Z., He N. (2013). Influence of nanoparticle shape, size, and surface functionalization on cellular uptake. J. Nanosci. Nanotechnol..

[6] Rubinsky B. (2007). Irreversible electroporation in medicine. Technol. Cancer Res. Treat..

[7] Zhang Y., Yu L.-C. (2008). Microinjection as a tool of mechanical delivery. Curr. Opin. Biotechnol..

[8] Pietroiusti A., Campagnolo L., Fadeel B. (2013). Interactions of engineered nanoparticles with organs protected by internal biological barriers. Small.

[9] Bode G.H., Coué G., Freese C., Pickl K.E., Sanchez-Purrà M., Albaiges B., Borrós S., van Winden E.C., Tziveleka L.-A., Sideratou Z. (2017). An in vitro and in vivo study of peptide-functionalized nanoparticles for brain targeting: The importance of selective blood–brain barrier uptake. Nanomedicine.

[10] Backes W.L. (2015). Passive Diffusion of Drugs across Membranes.

[11] Stillwell W. (2016). An Introduction to Biological Membranes: Composition, Structure and Function.

[12] Gouaux E., MacKinnon R. (2005). Principles of selective ion transport in channels and pumps. Science.

[13] Zhao F., Zhao Y., Liu Y., Chang X., Chen C., Zhao Y. (2011). Cellular uptake, intracellular trafficking, and cytotoxicity of nanomaterials. Small.

[14] Shang L., Nienhaus K., Nienhaus G.U. (2014). Engineered nanoparticles interacting with cells: Size matters. J. Nanobiotechnol..

[15] Liu Y., Li L., Li L., Zhou Z., Wang F., Xiong X., Zhou R., Huang Y. (2018). Programmed drug delivery system based on optimized “size decrease and hydrophilicity/hydrophobicity transformation” for enhanced hepatocellular carcinoma therapy of doxorubicin. Nanomed. Nanotechnol. Biol. Med..

[16] Abu Lila A.S., Kiwada H., Ishida T. (2013). The accelerated blood clearance (ABC) phenomenon: Clinical challenge and approaches to manage. J. Control. Release.

[17] Su Y., Wang L., Liang K., Liu M., Liu X., Song Y., Deng Y. (2018). The accelerated blood clearance phenomenon of PEGylated nanoemulsion upon cross administration with nanoemulsions modified with polyglycerin. Asian J. Pharm. Sci..

[18] Wu L., Zhang L., Shi G., Ni C. (2016). Zwitterionic pH/redox nanoparticles based on dextran as drug carriers for enhancing tumor intercellular uptake of doxorubicin. Mater. Sci. Eng. C.

[19] Chen D., Wang J., Wang Y., Zhang F., Dong X., Jiang L., Tang Y., Zhang H., Li W. (2018). Promoting Inter-/Intra-Cellular Process of Nanomedicine through its Physicochemical Properties Optimization. Curr. Drug Metab..

[20] Kumari S., Swetha M., Mayor S. (2010). Endocytosis unplugged: Multiple ways to enter the cell. Cell Res..

[21] Rabinovitch M. (1995). Professional and non-professional phagocytes: An introduction. Trends Cell Biol..

[22] Colucci-Guyon E., Niedergang F., Wallar B.J., Peng J., Alberts A.S., Chavrier P. (2005). A role for mammalian diaphanous-related formins in complement receptor (CR3)-mediated phagocytosis in macrophages. Curr. Biol..

[23] Swanson J.A. (2008). Shaping cups into phagosomes and macropinosomes. Nat. Rev. Mol. Cell Biol..

[24] Swanson J.A., Baer S.C. (1995). Phagocytosis by zippers and triggers. Trends Cell Biol..

[25] Doherty G.J., McMahon H.T. (2009). Mechanisms of endocytosis. Annu. Rev. Biochem..

[26] Lim J.P., Gleeson P.A. (2011). Macropinocytosis: An endocytic pathway for internalising large gulps. Immunol. Cell Biol..

[27] Swanson J.A., Watts C. (1995). Macropinocytosis. Trends Cell Biol..

[28] Conner S.D., Schmid S.L. (2003). Regulated portals of entry into the cell. Nature.

[29] Elkin S.R., Lakoduk A.M., Schmid S.L. (2016). Endocytic pathways and endosomal trafficking: A primer. Wien. Med. Wochenschr..

[30] Sahay G., Alakhova D.Y., Kabanov A.V. (2010). Endocytosis of nanomedicines. J. Control. Release.

[31] Pelkmans L., Helenius A. (2002). Endocytosis via caveolae. Traffic.

[32] Anderson R.G. (1998). The caveolae membrane system. Annu. Rev..

[33] Pelkmans L., Kartenbeck J., Helenius A. (2001). Caveolar endocytosis of simian virus 40 reveals a new two-step vesicular-transport pathway to the ER. Nat. Cell Biol..

[34] Schnitzer J.E., Liu J., Oh P. (1995). Endothelial caveolae have the molecular transport machinery for vesicle budding, docking, and fusion including VAMP, NSF, SNAP, annexins, and GTPases. J. Biol. Chem..

[35] Kiss A.L., Botos E. (2009). Endocytosis via caveolae: Alternative pathway with distinct cellular compartments to avoid lysosomal degradation?. J. Cell Mol. Med..

[36] Lamaze C., Dujeancourt A., Baba T., Lo C.G., Benmerah A., Dautry-Varsat A. (2001). Interleukin 2 receptors and detergent-resistant membrane domains define a clathrin-independent endocytic pathway. Mol. Cell..

[37] Mayor S., Pagano R.E. (2007). Pathways of clathrin-independent endocytosis. Nat. Rev. Mol. Cell Biol..

[38] Nabi I.R., Le P.U. (2003). Caveolae/raft-dependent endocytosis. J. Cell Biol..

[39] Almers W. (1990). Exocytosis. Annu. Rev. Physiol..

[40] Sakhtianchi R., Minchin R.F., Lee K.-B., Alkilany A.M., Serpooshan V., Mahmoudi M. (2013). Exocytosis of nanoparticles from cells: Role in cellular retention and toxicity. Adv. Colloid Interface Sci..

[41] Berg J., Tymoczko J., Stryer L. (2002). Biochemistry.

[42] Hall J.E. (2015). Guyton and Hall Textbook of Medical Physiology.

[43] Cooper G.M., Hausman R. (2000). A Molecular Approach: The Cell.

[44] Haque S., Pouton C.W., McIntosh M.P., Ascher D.B., Keizer D.W., Whittaker M., Kaminskas L.M. (2020). The impact of size and charge on the pulmonary pharmacokinetics and immunological response of the lungs to PLGA nanoparticles after intratracheal administration to rats. Nanomedicine.

[45] Wu M., Guo H., Liu L., Liu Y., Xie L. (2019). Size-dependent cellular uptake and localization profiles of silver nanoparticles. Int. J. Nanomed..

[46] Yue J., Feliciano T.J., Li W., Lee A., Odom T.W. (2017). Gold nanoparticle size and shape effects on cellular uptake and intracellular distribution of siRNA nanoconstructs. Bioconjug. Chem..

[47] Mohammad-Beigi H., Hayashi Y., Zeuthen C.M., Eskandari H., Scavenius C., Juul-Madsen K., Vorup-Jensen T., Enghild J.J., Sutherland D.S. (2020). Mapping and identification of soft corona proteins at nanoparticles and their impact on cellular association. Nat. Commun..

[48] Du X.-J., Wang J.-L., Iqbal S., Li H.-J., Cap Z.-T., Wang Y.-C., Du J.-Z., Wang J. (2018). The effect of surface charge on oral absorption of polymeric nanoparticles. Biomater. Sci..

[49] Bewersdorff T., Gruber A., Eravci M., Dumbani M., Klinger D., Haase A. (2019). Amphiphilic nanogels: Influence of surface hydrophobicity and cellular uptake. Int. J. Nanomed..

[50] Dalal C., Jana N.R. (2018). Galactose multivalency effect on the cell uptake mechanism of bioconjugated nanoparticles. J. Phys. Chem. C.

[51] Eshaghi B., Alsharif N., An X., Akiyama H., Brown K.A., Gummuluru S., Reinhard B.M. (2020). Stiffness of HIV-1 mimicking polymer nanoparticles modulates ganglioside-mediated cellular uptake and trafficking. Adv. Sci..

[52] Hoshyar N., Gray S., Han H., Bao G. (2016). The effect of nanoparticle size on in vivo pharmacokinetics and cellular interaction. Nanomedicine.

[53] Rejman J., Oberle V., Zuhorn I.S., Hoekstra D. (2004). Size-dependent internalization of particles via the pathways of clathrin-and caveolae-mediated endocytosis. Biochem. J..

[54] Sun Q., Ishii T., Kanehira K., Sato T., Taniguchi A. (2017). Uniform TiO_2_ nanoparticles induce apoptosis in epithelial cell lines in a size-dependent manner. Biomater. Sci..

[55] Yohan D., Cruje C., Lu X., Chithrani D.B. (2016). Size-dependent gold nanoparticle interaction at nano–micro interface using both monolayer and multilayer (tissue-like) cell models. Nano-micro Lett..

[56] Wong A.C., Wright D.W. (2016). Size-Dependent Cellular Uptake of DNA Functionalized Gold Nanoparticles. Small.

[57] Chithrani B.D., Ghazani A.A., Chan W.C. (2006). Determining the size and shape dependence of gold nanoparticle uptake into mammalian cells. Nano Lett..

[58] Shan Y., Ma S., Nie L., Shang X., Hao X., Tang Z., Wang H. (2011). Size-dependent endocytosis of single gold nanoparticles. Chem. Commun..

[59] Cho E.C., Au L., Zhang Q., Xia Y. (2010). The effects of size, shape, and surface functional group of gold nanostructures on their adsorption and internalization by cells. Small.

[60] Elbakry A., Wurster E.C., Zaky A., Liebl R., Schindler E., Bauer-Kreisel P., Blunk T., Rachel R., Goepferich A., Breunig M. (2012). Layer-by-Layer Coated Gold Nanoparticles: Size-Dependent Delivery of DNA into Cells. Small.

[61] Simons T.J. (1986). Passive transport and binding of lead by human red blood cells. J. Physiol..

[62] Xia Q., Zhang Y., Li Z., Hou X., Feng N. (2019). Red blood cell membrane-camouflaged nanoparticles: A novel drug delivery system for antitumor application. Acta Pharm. Sin. B.

[63] Rothen-Rutishauser B.M., Schürch S., Haenni B., Kapp N., Gehr P. (2006). Interaction of fine particles and nanoparticles with red blood cells visualized with advanced microscopic techniques. Environ. Sci. Technol..

[64] Li L., Xi W.-S., Su Q., Li Y., Yan G.-H., Liu Y., Wang H., Cao A. (2019). Unexpected Size Effect: The Interplay between Different-Sized Nanoparticles in Their Cellular Uptake. Small.

[65] Zhao Z., Ukidve A., Krishnan V., Mitragotri S. (2019). Effect of physicochemical and surface properties on in vivo fate of drug nanocarriers. Adv. Drug Deliv. Rev..

[66] Zhang D., Wei L., Zhong M., Xiao L., Li H.-W., Wang J. (2018). The morphology and surface charge-dependent cellular uptake efficiency of upconversion nanostructures revealed by single-particle optical microscopy. Chem. Sci..

[67] Xie X., Liao J., Shao X., Li Q., Lin Y. (2017). The Effect of shape on Cellular Uptake of Gold Nanoparticles in the forms of Stars, Rods, and Triangles. Sci. Rep..

[68] Da Silva-Candal A., Brown T., Krishnan V., Lopez-Loureiro I., Ávila-Gómez P., Pusuluri A., Pérez-Díaz A., Correa-Paz C., Hervella P., Castillo J. (2019). Shape effect in active targeting of nanoparticles to inflamed cerebral endothelium under static and flow conditions. J. Control. Release.

[69] Salatin S., Dizaj S.M., Yari Khosroushahi A. (2015). Effect of the surface modification, size, and shape on cellular uptake of nanoparticles. Cell Biol. Int..

[70] Huang X., Teng X., Chen D., Tang F., He J. (2010). The effect of the shape of mesoporous silica nanoparticles on cellular uptake and cell function. Biomaterials.

[71] Verma A., Stellacci F. (2010). Effect of surface properties on nanoparticle–cell interactions. Small.

[72] Truong N.P., Whittaker M.R., Mak C.W., Davis T.P. (2015). The importance of nanoparticle shape in cancer drug delivery. Expert Opin. Drug Deliv..

[73] Zhang K., Fang H., Chen Z., Taylor J.-S.A., Wooley K.L. (2008). Shape Effects of Nanoparticles Conjugated with Cell-Penetrating Peptides (HIV Tat PTD) on CHO Cell Uptake. Bioconjug. Chem..

[74] Park S.J. (2020). Protein–Nanoparticle Interaction: Corona Formation and Conformational Changes in Proteins on Nanoparticles. Int. J. Nanomed..

[75] Tekie F.S.M., Hajiramezanali M., Geramifar P., Raoufi M., Dinarvand R., Soleimani M., Atyabi F. (2020). Controlling evolution of protein corona: A prosperous approach to improve chitosan-based nanoparticle biodistribution and half-life. Sci. Rep..

[76] Francia V., Yang K., Deville S., Reker-Smit C., Nelissen I., Salvati A. (2019). Corona Composition Can Affect the Mechanisms Cells Use to Internalize Nanoparticles. ACS Nano.

[77] Lesniak A., Fenaroli F., Monopoli M.P., Åberg C., Dawson K.A., Salvati A. (2012). Effects of the presence or absence of a protein corona on silica nanoparticle uptake and impact on cells. ACS Nano.

[78] Hadjidemetriou M., Kostarelos K. (2017). Nanomedicine: Evolution of the nanoparticle corona. Nat. Nanotechnol..

[79] Zhdanov V.P., Cho N.-J. (2016). Kinetics of the formation of a protein corona around nanoparticles. Math. Biosci..

[80] Van Hong Nguyen B.-J.L. (2017). Protein corona: A new approach for nanomedicine design. Int. J. Nanomed..

[81] Monopoli M.P., Walczyk D., Campbell A., Elia G., Lynch I., Bombelli F.B., Dawson K.A. (2011). Physical-chemical aspects of protein corona: Relevance to in vitro and in vivo biological impacts of nanoparticles. J. Am. Chem. Soc..

[82] Lima T., Bernfur K., Vilanova M., Cedervall T. (2020). Understanding the Lipid and Protein Corona Formation on Different Sized Polymeric Nanoparticles. Sci. Rep..

[83] Hellstrand E., Lynch I., Andersson A., Drakenberg T., Dahlbäck B., Dawson K.A., Linse S., Cedervall T. (2009). Complete high-density lipoproteins in nanoparticle corona. FEBS J..

[84] Gustafson H.H., Holt-Casper D., Grainger D.W., Ghandehari H. (2015). Nanoparticle uptake: The phagocyte problem. Nano Today.

[85] Fam S.Y., Chee C.F., Yong C.Y., Ho K.L., Mariatulqabtiah A.R., Tan W.S. (2020). Stealth Coating of Nanoparticles in Drug-Delivery Systems. Nanomaterials.

[86] Blanco E., Shen H., Ferrari M. (2015). Principles of nanoparticle design for overcoming biological barriers to drug delivery. Nat. Biotechnol..

[87] Pozzi D., Colapicchioni V., Caracciolo G., Piovesana S., Capriotti A.L., Palchetti S., De Grossi S., Riccioli A., Amenitsch H., Laganà A. (2014). Effect of polyethyleneglycol (PEG) chain length on the bio–nano-interactions between PEGylated lipid nanoparticles and biological fluids: From nanostructure to uptake in cancer cells. Nanoscale.

[88] Salvati A., Pitek A.S., Monopoli M.P., Prapainop K., Bombelli F.B., Hristov D.R., Kelly P.M., Åberg C., Mahon E., Dawson K.A. (2013). Transferrin-functionalized nanoparticles lose their targeting capabilities when a biomolecule corona adsorbs on the surface. Nat. Nanotechnol..

[89] Jo D.H., Kim J.H., Lee T.G., Kim J.H. (2015). Size, surface charge, and shape determine therapeutic effects of nanoparticles on brain and retinal diseases. Nanomedicine.

[90] Ayala V., Herrera A.P., Latorre-Esteves M., Torres-Lugo M., Rinaldi C. (2013). Effect of surface charge on the colloidal stability and in vitro uptake of carboxymethyl dextran-coated iron oxide nanoparticles. J. Nanopart. Res..

[91] Bertoli F., Garry D., Monopoli M.P., Salvati A., Dawson K.A. (2016). The Intracellular Destiny of the Protein Corona: A Study on its Cellular Internalization and Evolution. ACS Nano.

[92] Gessner A., Lieske A., Paulke B.R., Müller R.H. (2003). Functional groups on polystyrene model nanoparticles: Influence on protein adsorption. J. Biomed. Mater. Res. A..

[93] Prozeller D., Pereira J., Simon J., Mailänder V., Morsbach S., Landfester K. (2019). Prevention of Dominant IgG Adsorption on Nanocarriers in IgG-Enriched Blood Plasma by Clusterin Precoating. Adv. Sci..

[94] Anderson C.R., Gnopo Y.D., Gambinossi F., Mylon S.E., Ferri J.K. (2018). Modulation of cell responses to Ag-(MeO2MA-co-OEGMA): Effects of nanoparticle surface hydrophobicity and serum proteins on cellular uptake and toxicity. J. Biomed. Mater. Res. A.

[95] Mahaling B., Katti D.S. (2016). Physicochemical properties of core–shell type nanoparticles govern their spatiotemporal biodistribution in the eye. Nanomedicine.

[96] Larson T.A., Joshi P.P., Sokolov K. (2012). Preventing protein adsorption and macrophage uptake of gold nanoparticles via a hydrophobic shield. ACS Nano.

[97] Moyano D.F., Saha K., Prakash G., Yan B., Kong H., Yazdani M., Rotello V.M. (2014). Fabrication of corona-free nanoparticles with tunable hydrophobicity. ACS Nano.

[98] Li Y., Chen X., Gu N. (2008). Computational investigation of interaction between nanoparticles and membranes: Hydrophobic/hydrophilic effect. J. Phys. Chem. B.

[99] Curtis E.M., Bahrami A.H., Weikl T.R., Hall C.K. (2015). Modeling nanoparticle wrapping or translocation in bilayer membranes. Nanoscale.

[100] Bai X., Wang S., Yan X., Zhou H., Zhan J., Liu S., Sharma V.K., Jiang G., Zhu H., Yan B. (2019). Regulation of Cell Uptake and Cytotoxicity by Nanoparticle Core under the Controlled Shape, Size, and Surface Chemistries. ACS Nano.

[101] Richards D.A., Maruani A., Chudasama V. (2017). Antibody fragments as nanoparticle targeting ligands: A step in the right direction. Chem. Sci..

[102] Rodriguez P.L., Harada T., Christian D.A., Pantano D.A., Tsai R.K., Discher D.E. (2013). Minimal” Self” peptides that inhibit phagocytic clearance and enhance delivery of nanoparticles. Science.

[103] Parodi A., Quattrocchi N., Van De Ven A.L., Chiappini C., Evangelopoulos M., Martinez J.O., Brown B.S., Khaled S.Z., Yazdi I.K., Enzo M.V. (2013). Synthetic nanoparticles functionalized with biomimetic leukocyte membranes possess cell-like functions. Nat. Nanotechnol..

[104] Gradishar W.J. (2006). Albumin-bound paclitaxel: A next-generation taxane. Expert Opin. Pharmacother..

[105] Figueroa S.M., Fleischmann D., Beck S., Goepferich A. (2020). The effect of ligand mobility on the cellular interaction of multivalent nanoparticles. Macromol. Biosci..

[106] Li L., Zhang Y., Wang J. (2017). Effects of ligand distribution on receptor-diffusion-mediated cellular uptake of nanoparticles. R. Soc. Open Sci..

[107] Schubertová V., Martinez-Veracoechea F.J., Vácha R. (2015). Influence of ligand distribution on uptake efficiency. Soft Matter.

[108] Donahue N.D., Acar H., Wilhelm S. (2019). Concepts of nanoparticle cellular uptake, intracellular trafficking, and kinetics in nanomedicine. Adv. Drug Deliv. Rev..

[109] Chen L., Li X., Zhang Y., Chen T., Xiao S., Liang H. (2018). Morphological and mechanical determinants of cellular uptake of deformable nanoparticles. Nanoscale.

[110] Sun J., Zhang J., Wang Q., Feng D., Liu Q., Yin D., Xu Y., Wei B., Ding X., Shi X. (2015). Tunable rigidity of (polymeric core)-(lipid shell) nanoparticles for regulated cellular uptake. Adv. Mater..

[111] Wei C., Wei R., Jiang G., Jia Y., Lou H., Yang Z., Luo D., Huang Q., Xu S., Yang X. (2019). Mechnical cues modulate ceullar uptake of nanoparticles in cancer via clathrin-mediated and caveolae-mediated endocytosis pathways. Nanomedicine.

[112] Guo P., Liu D., Subramanyam K., Wang B., Yang J., Huang J., Auguste D.T., Moses M.A. (2018). Nanoparticle elasticity directs tumor uptake. Nat. Commun..

[113] Nasrollahzadeh M., Sajadi M.S., Atarod M., Sajjadi M., Isaabadi Z. (2019). An Introduction to Green Nanotechnology.

[114] Hui Y., Wibowo D., Liu Y., Ran R., Wang H.-F., Seth A., Middelberg A.P., Zhao C.-X. (2018). Understanding the effects of nanocapsular mechanical property on passive and active tumor targeting. ACS Nano.

[115] Zhao J., Lu H., Yao Y., Ganda S., Stenzel M.H. (2018). Length vs. stiffness: Which plays a dominant role in the cellular uptake of fructose-based rod-like micelles by breast cancer cells in 2D and 3D cell culture models?. J. Mater. Chem. B.

[116] Anselmo A.C., Zhang M., Kumar S., Vogus D.R., Menegatti S., Helgeson M.E., Mitragotri S. (2015). Elasticity of nanoparticles influences their blood circulation, phagocytosis, endocytosis, and targeting. ACS Nano.

[117] Wang S., Guo H., Li Y., Li X. (2019). Penetration of nanoparticles across a lipid bilayer: Effects of particle stiffness and surface hydrophobicity. Nanoscale.

